# Geographical, genetic and functional diversity of antiretroviral host factor TRIMCyp in cynomolgus macaque (*Macaca fascicularis*)

**DOI:** 10.1099/vir.0.038075-0

**Published:** 2012-03

**Authors:** Akatsuki Saito, Ken Kono, Masako Nomaguchi, Yasuhiro Yasutomi, Akio Adachi, Tatsuo Shioda, Hirofumi Akari, Emi E. Nakayama

**Affiliations:** 1 Primate Research Institute, Kyoto University, Inuyama 484-8506, Japan; 2 Department of Viral Infections, Research Institute for Microbial Diseases, Osaka University, Suita 565-0871, Japan; 3 Department of Microbiology, Institute of Health Biosciences, The University of Tokushima Graduate School, Tokushima 770-8503, Japan; 4 Tsukuba Primate Research Center, National Institute of Biomedical Innovation, Tsukuba 305-0843, Japan

## Abstract

The antiretroviral factor tripartite motif protein 5 (*TRIM5*) gene-derived isoform (TRIMCyp) has been found in at least three species of Old World monkey: rhesus (*Macaca mulatta*), pig-tailed (*Macaca nemestrina*) and cynomolgus (*Macaca fascicularis*) macaques. Although the frequency of TRIMCyp has been well studied in rhesus and pig-tailed macaques, the frequency and prevalence of TRIMCyp in cynomolgus macaques remain to be definitively elucidated. Here, the geographical and genetic diversity of TRIM5α/TRIMCyp in cynomolgus macaques was studied in comparison with their anti-lentiviral activity. It was found that the frequency of TRIMCyp in a population in the Philippines was significantly higher than those in Indonesian and Malaysian populations. Major and minor haplotypes of cynomolgus macaque TRIMCyp with single nucleotide polymorphisms in the cyclophilin A domain were also found. The functional significance of the polymorphism in TRIMCyp was examined, and it was demonstrated that the major haplotype of TRIMCyp suppressed human immunodeficiency virus type 1 (HIV-1) but not HIV-2, whilst the minor haplotype of TRIMCyp suppressed HIV-2 but not HIV-1. The major haplotype of TRIMCyp did not restrict a monkey-tropic HIV-1 clone, NL-DT5R, which contains a capsid with the simian immunodeficiency virus-derived loop between α-helices 4 and 5 and the entire *vif* gene. These results indicate that polymorphisms of TRIMCyp affect its anti-lentiviral activity. Overall, the results of this study will help our understanding of the genetic background of cynomolgus macaque TRIMCyp, as well as the host factors composing species barriers of primate lentiviruses.

## Introduction

Human immunodeficiency virus type 1 (HIV-1) barely replicates in Old World monkeys such as cynomolgus macaques (CMs; *Macaca fascicularis*) and rhesus macaques (RMs; *Macaca mulatta*). This species barrier has long hampered the use of Old World monkeys for human immunodeficiency virus type 1 (HIV-1) research. Recently, a number of intrinsic anti-HIV-1 cellular factors, including tripartite motif protein 5α (TRIM5α), cyclophilin A (CypA), the apolipoprotein B mRNA-editing enzyme catalytic polypeptide-like 3 (APOBEC3) family and tetherin were identified in Old World monkey cells (Nomaguchi *et al.*, 2008; Sauter *et al.*, 2010). Of these factors, TRIM5α was found to strongly suppress HIV-1 replication, mainly by affecting the virus disassembly step, resulting in a decrease in reverse-transcription products (Nakayama & Shioda, 2010; Stremlau *et al.*, 2004). TRIM5α contains a RING domain, a B-box domain, a coiled-coil domain and a PRYSPRY (B30.2) domain. Importantly, the PRYSPRY domain recognizes the capsid of incoming retroviruses, leading to post-entry restriction of infection. RM and CM TRIM5α restrict HIV-1 but not simian immunodeficiency virus isolated from an infected rhesus macaque (SIVmac) (Nakayama *et al.*, 2005; Stremlau *et al.*, 2004; Yap *et al.*, 2004). In the case of HIV-2 infection, viruses carrying proline (P) at aa 120 of the capsid protein are sensitive to CM TRIM5α, whereas those with either alanine or glutamine (Q) are resistant (Song *et al.*, 2007). Both CM TRIM5α-sensitive and -resistant HIV-2 strains are restricted by RM TRIM5α, and three amino acid residues – threonine (T), phenylalanine (F) and P at aa 339, 340 and 341, respectively – of RM TRIM5α are important for restricting particular HIV-2 strains, which are still resistant to CM TRIM5α (Kono *et al.*, 2008). It is also known that TRIM5α exhibits a high degree of sequence variation, even within macaque species. In some individual RMs, the TFP residues at aa 339–341 of TRIM5α are replaced with a single Q (Newman *et al.*, 2006) and this TFP→Q polymorphism affects the anti-lentiviral activity of RM TRIM5α (Kirmaier *et al.*, 2010).

Although pig-tailed macaques (PMs; *Macaca nemestrina*) have long been thought to exhibit a higher susceptibility to HIV-1 infection than RMs and CMs (Agy *et al.*, 1992), the underlying mechanism determining this difference remained unclear. Recently, a TRIM5–CypA chimeric protein, referred to as TRIMCyp, was discovered in PMs, and the monkeys exclusively expressed TRIMCyp but not TRIM5α (Brennan *et al.*, 2008; Liao *et al.*, 2007). TRIMCyp is an alternatively spliced isoform of the *TRIM5* gene in which the PRYSPRY domain of TRIM5α is replaced with a retrotransposed *CypA* gene. The retrotransposition of the *CypA* sequence in the 3′ UTR of the *TRIM5* gene correlates with a single nucleotide polymorphism (SNP) at the exon 7 splice-acceptor site, leading to skipping of exons 7 and 8 encoding the PRYSPRY domain and splicing to the *CypA* insertion. Thus, the presence or absence of the *CypA* sequence in the 3′ UTR results in expression of TRIMCyp or TRIM5α, respectively.

*In vitro* analyses demonstrated that cells expressing PM TRIMCyp restricted HIV-2 but not HIV-1 infection (Brennan *et al.*, 2008; Liao *et al.*, 2007), suggesting that the characteristic isoform of the *TRIM5* gene in PMs may be one of the reasons for their greater susceptibility to HIV-1 infection. Furthermore, TRIMCyp was also identified in some individual RMs and CMs (Brennan *et al.*, 2008; Newman *et al.*, 2008; Wilson *et al.*, 2008a). RM TRIMCyp, as well as that of PMs, is unable to restrict HIV-1 infection (Wilson *et al.*, 2008a). This report also showed that the frequency of TRIMCyp in Indian RMs was approximately 25 %, whilst it was not found in the Chinese RM population, suggesting a geographical deviation in the frequency of TRIMCyp (Wilson *et al.*, 2008a). In the case of CMs, although the existence of TRIMCyp has been reported (Brennan *et al.*, 2008), the allele frequency, geographical distribution and relevance in antiviral activity of TRIMCyp remain to be elucidated. As the *TRIM5* gene-related factors are expected to have an impact on the replication of retroviruses, information about the genetic background of CM TRIMCyp will contribute to our understanding of host factors composing the species barrier. In the present study, we studied the geographical, genetic and functional diversity of CM TRIMCyp originating from South-West Asia (Indonesia, Malaysia and the Philippines). We showed a geographical deviation in the frequency of TRIMCyp. Moreover, we found SNPs in CM TRIMCyp and analysed their impact on the anti-lentiviral functions, including their effect against HIV-1, HIV-2, SIVmac and monkey-tropic HIV-1 (HIV-1mt).

## Results

### Geographical deviation in the frequency of CM TRIMCyp

**Fig. 1.**
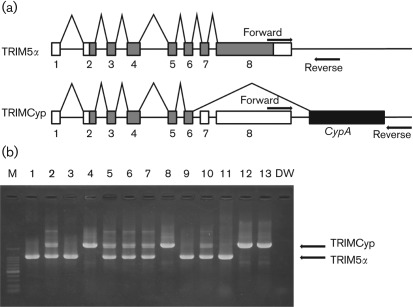
Determination of *CypA* insertion. (a) Diagram indicating the splicing of TRIM5α or TRIMCyp. Non-coding and coding exons (numbered) and *CypA* sequences are indicated as open, shaded and filled boxes, respectively. The primers used in this study are indicated by arrows. (b) Genomic DNA was extracted from PBMCs. To test for *CypA* insertion, the 3′ region of the *TRIM5* gene was amplified by PCR with primers spanning the 3′ UTR and the putative *CypA* insertion. DW, Distilled water control.

Initially, we analysed the frequencies of TRIM5α and TRIMCyp genotypes in 126 CMs originating from three different regions – Indonesia, Malaysia and the Philippines – using a PCR-based assay designed to differentiate between the presence and absence of the *CypA* insertion (Fig. 1a) (Wilson *et al.*, 2008a). Insertion of the *CypA* gene in TRIMCyp resulted in a PCR product larger than the expected size for TRIM5α (Fig. 1b).

**Fig. 2.**
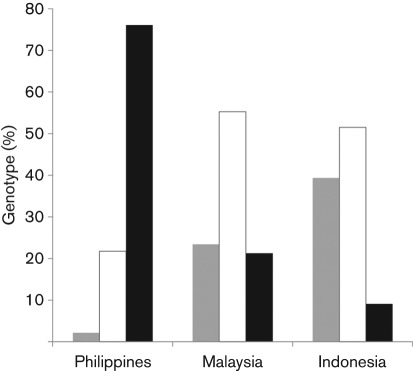
Frequency of individuals having each *TRIM5* genotype. The percentages of TRIM5α homozygotes and heterozygotes and TRIMCyp homozygotes in each population were calculated. Grey bars, TRIM5α homozygote; white bars, heterozygote; black bars, TRIMCyp homozygote.

As shown in Table 1, 35 of the 46 Philippine individuals were homozygous for TRIMCyp, ten were heterozygous and only one was homozygous for TRIM5α. In contrast, only three of the 33 Indonesian individuals were homozygous for TRIMCyp, 17 were heterozygous and 13 were homozygous for TRIM5α. Interestingly, the Malaysian population was of intermediate proportions: ten TRIMCyp homozygotes, 26 heterozygotes and 11 TRIM5α homozygotes. As shown in Fig. 2, the percentages of individuals having each *TRIM5* genotype indicated that the frequency of TRIMCyp homozygotes in Malaysian CMs was twice that in Indonesian CMs. In contrast, the frequency of TRIM5α homozygotes in Indonesian CMs was twice that in Malaysian CMs. Taken together, the calculated allele frequencies of TRIMCyp in the Philippine, Indonesian and Malaysian CM populations were 87.0, 34.8 and 48.9 %, respectively (Table 1). Statistical analyses using a χ^2^ test followed by Bonferroni correction demonstrated that the frequency of TRIMCyp in the Philippine population was significantly higher than that in the Indonesian (*P*<0.0001) and Malaysian (*P*<0.0001) populations. In contrast, there was no significant difference between the Indonesian and Malaysian populations (*P* = 0.2295).

**Table 1.** Frequencies of TRIMCyp alleles in CM populations

Origin	No. of animals	Genotype (no. of animals)			Allele frequency (%)	
		TRIM5α homozygote	TRIM5α/TRIMCyp heterozygote	TRIMCyp homozygote	TRIM5α	TRIMCyp
Philippines	46	1	10	35	13.0	87.0
Malaysia	47	11	26	10	51.1	48.9
Indonesia	33	13	17	3	65.2	34.8

It should be noted that our method failed to distinguish homozygotes from hemizygotes, especially when the subjects exhibited no polymorphisms in the *TRIM5* gene. However, hemizygosity for the *TRIM5* gene is highly unlikely for the following reasons: (i) the *TRIM5* gene is on an autosomal chromosome, (ii) there is no precedent of deletion of the *TRIM5* gene in humans or primates, and (iii) all of the three CM populations in Table 1 are in Hardy–Weinberg equilibrium for *TRIM5* genotypes.

### Polymorphisms in the *CypA* domain of CM TRIMCyp

Previously, it was reported that aa 357 of CM TRIMCyp, corresponding to aa 54 counting from the methionine of CypA, was arginine (R) (Brennan *et al.*, 2008). Subsequently, Ylinen *et al.* (2010) reported another allele of CM TRIMCyp encoding histidine (H) at this position. To determine the frequency of this R→H polymorphism, we examined 34 TRIM5α/TRIMCyp heterozygotes and 30 TRIMCyp homozygotes for sequence variations in the CypA domain. The results showed that there was no TRIMCyp allele encoding R at position 357 (Cyp 54R). All 94 CM chromosomes carrying the TRIMCyp gene encoded TRIMCyp with H at this position. This result was consistent with the results reported recently by Dietrich *et al.* (2011).

Dietrich *et al.* (2011) also reported CM TRIMCyp polymorphisms at aa 369 and 446, corresponding to aa 66 and 143 in the CypA domain, respectively. Both Brennan *et al.* (2008) and Ylinen *et al.* (2010) reported that aa 369 (Cyp66) and 446 (Cyp143) are aspartic acid (D) and lysine (K), respectively (denoted as the DK haplotype), whilst Dietrich *et al.* (2011) showed the presence of another haplotype encoding asparagine (N) and glutamic acid (E) at positions 369 (Cyp66) and 446 (Cyp143), respectively (denoted as the NE haplotype). Our results showed that 12 CM chromosomes carried TRIMCyp with the NE haplotype, whilst the remaining 82 TRIMCyp were all the DK haplotype (Table 2). Residues 369N (Cyp 66N) and 446E (Cyp 143E) were also found in PM and RM TRIMCyps, and the CypA portion of the NE haplotype of CM TRIMCyp has the same amino acid sequence as RM TRIMCyp (GenBank accession no. EU157763). These results indicate that the previously recognized interspecies variations of the CypA sequence of TRIMCyp were in fact intraspecies variation within CMs. With respect to the geographical distribution of these haplotypes, we found no significant deviation in the frequencies of the haplotypes among the three origins (Table 2).

**Table 2.** Frequencies of DK and NE haplotypes in CM TRIMCyps

Origin	No. of animals	Genotype (no. of chromosomes)				Frequency (%)	
		TRIM5α/TRIMCyp heterozygote*		TRIMCyp homozygote†		DK	NE
		DK	NE	DK	NE		
Philippines	28	6	1	36	6	85.7	14.3
Malaysia	21	14	1	10	2	88.9	11.1
Indonesia	15	12	0	4	2	88.9	11.1

* Haplotypes were determined by direct sequencing of the PCR products.

† Haplotypes were inferred by maximum-likelihood estimation using the results of direct sequencing of the PCR products.

### Polymorphisms in the RING, B-box, coiled-coil, linker and PRYSPRY domains of CM TRIM5α and TRIMCyp

**Fig. 3.**
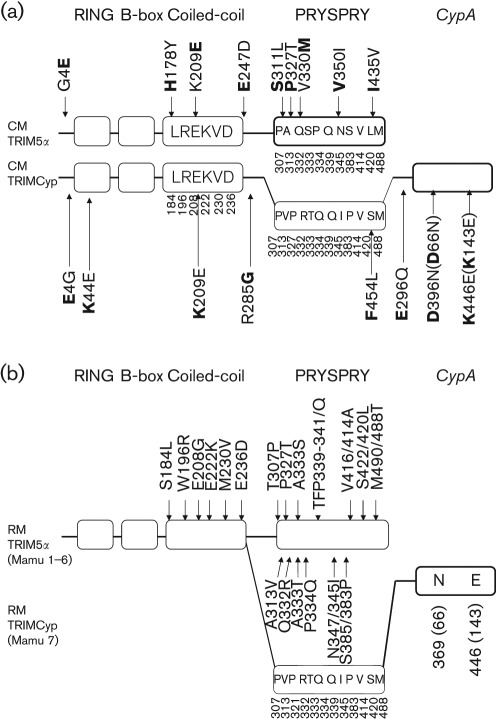
Sequence variations in TRIM5α and TRIMCyp. (a) Sequence variations in CM TRIM5α and TRIMCyp. The RING, B-box, coiled-coil, PRYSPRY and *CypA* domains of CM TRIM5α and TRIMCyp are indicated by open boxes. The box with thin lines shows exons 7 and 8 of the TRIMCyp gene, which is absent from the mRNA. Polymorphisms are shown outside the boxes, with downward and upward arrows indicating the polymorphisms observed among TRIM5α homozygotes and TRIMCyp homozygotes, respectively. Amino acid residues found in HSC-F cells are shown in front of the amino acid positions, followed by the observed polymorphisms. Major alleles are shown in bold. Numbers in parentheses indicate amino acid positions counting from the initiation methionine codon of the *CypA* ORF. Amino acid residues in the boxes are polymorphic in the RM *TRIM5* gene but lack polymorphism in CM TRIM5α or TRIMCyp. Positions of these amino acid residues are shown below the boxes. (b) Sequence variations of RM TRIM5α (Mamu 1–6) and TRIMCyp (Mamu 7). Downward and upward arrows indicate the polymorphisms observed in TRIM5α and TRIMCyp, respectively. Amino acid residues in boxes indicate those of RM TRIMCyp. Positions of these amino acid residues are shown below the boxes.

To identify polymorphisms that are in possible linkage disequilibrium with either the DK or NE haplotype in regions other than the CypA domain, we determined nucleotide sequences of TRIM5α and TRIMCyp cDNAs encoding the RING, B-box, coiled-coil and linker domains of six TRIMCyp homozygotes (three homozygotes of the DK haplotype and three heterozygotes for the DK and NE haplotypes) and three TRIM5α homozygotes (see Supplementary Table S1, available in JGV Online). We found polymorphisms at positions 4 [E→glycine (G)] in the N-terminal region, 44 (K→E) in the RING domain, 178 [H→tyrosine (Y)] and 209 (K→E) in the coiled-coil domain, and 247 (E→D) and 285 (G→R) in the linker domain (Fig. 3a). We found only one chromosome for minor allele 4G, two for 44E, four for 178Y, nine for 209E, five for 247D and four for 285R among 18 chromosomes from the six TRIMCyp homozygotes and three TRIM5α homozygotes. Among the six TRIMCyp homozygotes, we also found three E→Q substitutions at aa 296, which was present in TRIMCyp but absent from TRIM5α. There was no polymorphism that showed strong linkage disequilibrium with either the DK or NE haplotype except for G285R. The NE haplotype tended to link with 285G, although several DK haplotypes also linked with 285G (Supplementary Table S1). The numbers of polymorphic positions were relatively small among CMs compared with RMs (Fig. 3b). It is known that the coiled-coil region of *TRIM5* genes shows a high degree of genetic diversity in RMs (Johnson & Sawyer, 2009). In contrast, the coiled-coil domain of CM TRIM5α and TRIMCyp showed no polymorphism at aa 184, 196, 208, 222, 230 and 236, which were all highly polymorphic in RMs (Newman *et al.*, 2006). These results suggest that the evolutionary pressures targeting the coiled-coil domain of the *TRIM5* gene were weaker in CMs than in RMs.

We also determined the nucleotide sequences of exon 8 encoding the PRYSPRY domain, of 12 TRIM5α homozygotes including the three TRIM5α homozygotes analysed above (see Supplementary Table S2, available in JGV Online). We found polymorphisms at aa 311 [serine (S)→leucine (L)], 327 (P→T), 330 [valine (V)→methionine (M)], 350 [V→isoleucine (I)] and 435 (I→V) in the PRYSPRY domain (Fig. 3a). Among the 12 TRIM5α homozygotes, we did not find a TFP allele at aa 339–341, which is a major determinant for different virus specificity between CM and RM TRIM5αs (Kono *et al.*, 2008) and is also critical for SIV from sooty mangabeys (SIVsm) (Kirmaier *et al.*, 2010) and SIVmac (Lim *et al.*, 2010) restriction by RM TRIM5α. We found only one chromosome for minor allele 311L, one for 327T, one for 350I and four for 435V among 11 TRIM5α homozygotes. We previously cloned CM TRIM5α cDNA from HSC-F cells (GenBank accession no. AB210052) (Nakayama *et al.*, 2005) and found that it contained 330V; however, all of the sequences determined in the present study showed M at this position. In contrast, exon 8 of the TRIMCyp gene of seven TRIMCyp homozygotes (all were heterozygotes for the DK and NE haplotypes), which encoded the PRYSPRY domain but was absent from the mRNA due to splicing, showed a uniform sequence identical to that of the Mamu 7 haplotype of RMs (307P, 313V, 327P, 332R, 333T, 334Q, 339Q, 345I, 383P, 414V, 420S and 488M). We only found one F→L substitution at position 454 among the seven TRIMCyp homozygotes. The Mamu 7 sequence is thus likely to be an ancient prototype sequence of TRIMCyp before the separation of CMs from RMs.

### Anti-lentiviral activity of CM TRIMCyps

**Fig. 4.**
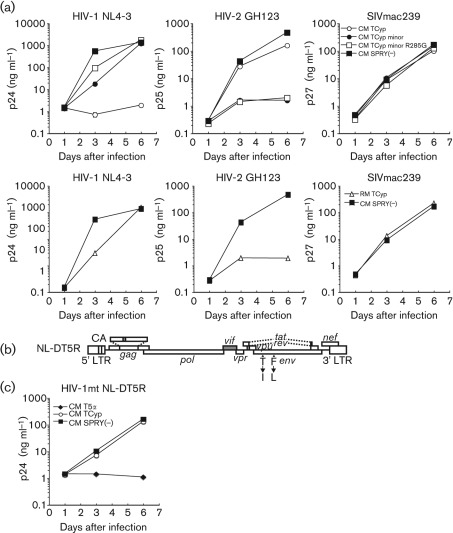
Anti-lentiviral activity of various CM TRIMCyp. (a) MT4 cells were infected with recombinant SeV expressing CM TRIMCyp-major (DK) (CM TCyp; ○), CM TRIMCyp-minor (NE) (CM TCyp minor; •), CM TRIMCyp-minor R285G (CM TCyp minor R285G; □), CM SPRY (−) (▪) or RM TRIMCyp (RM TCyp; ▵). Data for CM SPRY (−) in the upper and lower panels were identical. Nine hours after infection, cells were superinfected with HIV-1 NL4-3, HIV-2 GH123 or SIVmac239. Culture supernatants were assayed separately for levels of p24, p25 or p27. (b) Structure of HIV-1mt NL-DT5R used in the experiment shown in Fig. 3(c). Open boxes denote HIV-1 (NL4-3) and shaded boxes denote SIVmac239 sequences. (c) MT4 cells were infected with recombinant SeV expressing CM TRIM5α (CM T5α; ⧫), CM TRIMCyp-major (DK) (CM TCyp; ○) or CM SPRY (−) (▪). Nine hours after infection, cells were superinfected with HIV-1mt NL-DT5R. Culture supernatants were assayed separately for levels of p24. Error bars show actual fluctuations between duplicate samples. Data from a representative of three (a) or two (c) independent experiments are shown.

To elucidate the impact of CM TRIMCyp and its SNPs on the anti-lentiviral activity, we constructed a recombinant Sendai virus (SeV) expressing a series of TRIM5α/TRIMCyp: CM TRIM5α, the DK and NE haplotypes of the CM TRIMCyp [CM TRIMCyp-major (DK) and CM TRIMCyp-minor (NE)], CM SPRY (−) in which the PRYSPRY domain was deleted as a negative control for functional TRIM5α and TRIMCyp, and an RM TRIMCyp. We also constructed a recombinant SeV expressing a CM TRIMCyp-minor (NE) carrying G at position 285 (CM TRIMCyp-minor R285G), as the NE haplotype seemed to be in linkage disequilibrium with G at this position (Supplementary Table S1). As shown in Fig. 4[(a), upper panels], TRIMCyp-major (DK) completely restricted HIV-1 NL4-3, weakly restricted HIV-2 GH123 and failed to restrict SIVmac239. In contrast, TRIMCyp-minor (NE) and TRIMCyp-minor R285G inefficiently restricted HIV-1 NL4-3, barely restricted SIVmac239 and completely restricted HIV-2 GH123. These results indicated that the sequence variations in CM TRIMCyp greatly altered the spectrum of its anti-lentiviral activity. It should be noted that HIV-1 NL4-3 attained slightly higher titres at day 3 in cells expressing TRIMCyp-minor R285G than in those expressing TRIMCyp-minor (NE). The difference was small but reproducible in six independent experiments. This result indicated that aa 285 of TRIMCyp also affected its antiviral activity. In the case of RMs, in which the CypA domain of TRIMCyp has the same amino acid sequence as CM TRIMCyp-minor (NE), RM TRIMCyp showed the same spectrum of anti-lentiviral activity as CM TRIMCyp-minor (NE) (Fig. 4a, lower panels), consistent with previous reports (Price *et al.*, 2009; Wilson *et al.*, 2008a).

Finally, we examined whether HIV-1mt NL-DT5R (Kamada *et al.*, 2006) could evade restriction by CM TRIM5α/TRIMCyp. HIV-1mt possesses core antigen (CA) with the SIVmac239-derived loop between α-helices 4 and 5 (L4/5), which corresponds to a CypA-binding loop of HIV-1, the entire SIVmac239 *vif* gene and two non-synonymous substitutions in the *env* gene (Fig. 4b). As shown in Fig. 4(c), NL-DT5R was restricted by TRIM5α but completely evaded restriction by CM TRIMCyp-major (DK), suggesting that replacement of the CypA-binding loop of HIV-1 CA with the corresponding SIVmac239-derived sequence was sufficient to render HIV-1 resistant to the major haplotype of CM TRIMCyp but not TRIM5α.

## Discussion

In the present study, we analysed the geographical, genetic and functional diversity of CM TRIMCyp and found: (i) a clear geographical deviation in the frequency of TRIMCyp, (ii) no typical geographical deviation in the frequency of the DK/NE haplotypes in the CypA domain, and (iii) sequence variations in the CypA domain of CM TRIMCyp, which greatly altered the spectrum of its anti-lentiviral activity.

We first demonstrated that the allele frequency of TRIMCyp in CMs from the Philippines was significantly higher than those in Indonesian and Malaysian CMs. It is possible that some pathogen(s) resistant to the antiviral effect of either TRIM5α or TRIMCyp may contribute to this deviation as a selective pressure. As primate lentiviruses such as HIV-1 and SIV originated in African primates, it is unlikely that these viruses could contribute directly to this deviation, and some exogenous and endogenous retroviruses may thus play a critical role in this selection. Alternatively, it is possible that this deviation could come from bottleneck effects. It is estimated that the Philippine CMs were derived from Indonesian CM stocks via sea rafting or terrestrial access through Borneo during periods of low sea level in South-East Asia around 110 000 years ago (Abegg & Thierry, 2002; Blancher *et al.*, 2008; Kita *et al.*, 2009). Furthermore, phylogenetic analyses of mitochondrial DNA sequences of four CM populations distributed in South-East Asia suggested that Philippine CMs were derived from the small founding populations of Indonesian CMs, resulting in low genetic and nucleotide diversities (Blancher *et al.*, 2008). Importantly, however, as the Philippine CMs involved in this study at least originated from Luzon and Mindanao, the results in this study may reflect the frequency of TRIMCyp in Philippine CMs as a whole, but do not represent local TRIMCyp distribution. In addition, hybridization with RMs may affect the prevalence of TRIMCyp. As Chinese RMs have been reported to have a low frequency of TRIMCyp (Newman *et al.*, 2008; Wilson *et al.*, 2008a), it is possible that interspecies mating with Chinese RMs might result in a lower prevalence of TRIMCyp in the Malaysian and Indonesian populations. In any case, it will be of great interest to determine the allele frequency of TRIMCyp in wild CMs to confirm whether our results reflect the observations in nature.

It is worth noting that the habitat of PMs is close to that of CMs, and in fact both species inhabit Indonesia; however, PMs reportedly express TRIMCyp but not TRIM5α (Brennan *et al.*, 2008; Liao *et al.*, 2007). In contrast, the allele frequency of TRIMCyp in Indonesian CMs was shown to be markedly lower (Table 1). This discrepancy in frequency of TRIMCyp between PMs and CMs suggests that the two species have independently evolved antiretroviral factors to counteract some pathogen(s) existing in their habitats. It is possible that unidentified co-factors that interact with TRIM5α/TRIMCyp may have a role in this discrepancy. Alternatively, the pathogen(s) could develop severe diseases in either monkey species. In the case of RMs, whilst the allele frequency of TRIMCyp was approximately 25 % in the Indian population, TRIMCyp was not detected in the Chinese population (Wilson *et al.*, 2008a). Although the precise reason(s) for these geographical deviations in CMs and RMs is still unknown, it is reasonable to speculate that the possible pathogens, including exogenous and endogenous retroviruses, are/were heterogeneously disseminated, depending on their habitats.

The amino acid sequence of the CypA domain of our CM TRIMCyp-major (DK) is identical to that of Mafa TRIMCyp2 cloned by Ylinen *et al.* (2010); thus, CM TRIMCyp-major (DK) showed almost identical antiviral properties to those of Mafa TRIMCyp2. However, CM TRIMCyp-major (DK) slightly restricted HIV-2 GH123, although Mafa TRIMCyp2 failed to restrict HIV-2 ROD. This discrepancy is possibly due to differences in assays; Ylinen and co-workers performed a single-round infection assay using replication-incompetent virus, whereas we performed a multiple-round replication assay using replication-competent virus and thus our assay could detect weak restriction activities. It is also possible that differences in HIV-2 strains or TRIMCyp amino acid differences outside the CypA domain could affect the result.

In the case of CM TRIMCyp-minor (NE), the amino acid sequence of the CypA domain was identical to that of RM TRIMCyp, and antiviral properties of CM TRIMCyp-minor (NE) were the same as those of RM TRIMCyp. In addition, exon 8 of both TRIMCyp genes showed a uniform sequence, identical to that of the Mamu 7 haplotype of RMs. Exon 8 of TRIMCyp would have been free from selection pressures, as it is absent from the mRNA due to splicing, and the ancestral sequences in exon 8 would have been preserved. Taken together, it is reasonable to speculate that this minor haplotype of CM TRIMCyp was the ancestor when CMs separated from RMs, and the major haplotype of CM TRIMCyp has arisen due to a specific evolutionary pressure on CMs. It should be noted that CM TRIM5α has Q at aa 339, where RM TRIM5α has a Q→TFP polymorphism. This Q→TFP polymorphism in the PRYSPRY domain also altered the spectrum of anti-lentiviral activity of TRIM5α (Kirmaier *et al.*, 2010; Kono *et al.*, 2008; Lim *et al.*, 2010; Wilson *et al.*, 2008b). Therefore, it is tempting to speculate that the selection pressure in CMs drove amplification and diversification in TRIMCyp, whilst that in RMs drove diversification of the PRYSPRY domain of TRIM5α.

In parallel with our study, Dietrich *et al.* (2011) recently reported the prevalence and functional diversity of TRIMCyp in CMs. They analysed populations from Indonesia, Indochina, Mauritius and the Philippines, and found that TRIMCyp was present in populations from Indonesia, Indochina and the Philippines, but not in populations from Mauritius. As they mentioned, the low genetic diversity, probably due to founder effects, may have led to the absence of TRIMCyp in the Mauritian population. In contrast, the small number of animals analysed may have resulted in the absence of TRIM5α in their Philippine population. They also analysed the effects of DK→NE substitution in CM TRIMCyp on antiretroviral activity by mutagenesis techniques. Furthermore, they found a unique individual with the DE haplotype in the CypA domain of TRIMCyp, whilst we did not identify such a haplotype in our study. Their results were essentially in accordance with ours, and we further demonstrated that Philippine CMs possessed TRIM5α as well as TRIMCyp, suggesting that maintenance of both TRIM5α and TRIMCyp in the CM population is beneficial to counteract challenges by retroviruses that are susceptible to TRIM5α and by those susceptible to TRIMCyp. Consistent with this, Reynolds *et al.* (2011) demonstrated that heterozygotes of RMs with TRIM5α and TRIMCyp showed higher resistance to repeated intrarectal challenge of SIVsmE660 compared with homozygotes for TRIM5α or TRIMCyp. Interestingly, this different outcome was not observed in the case of intrarectal challenge with SIVmac239. As RM TRIMCyp restricts SIVsm but not SIVmac (Kirmaier *et al.*, 2010), the combination of TRIM5α and TRIMCyp may function more efficiently as an antiviral factor against SIVsm.

We saw a small difference in anti-HIV-1 activity between CM TRIMCyp-minor (NE) and TRIMCyp-minor R285G. Dietrich *et al.* (2011) suggested that either of two polymorphic amino acid residues, K209E and R285G, might be responsible for attenuated anti-feline immunodeficiency virus activity of a certain haplotype of CM TRIMCyp. Our CM TRIMCyp-minor (NE) had K at aa 209, and an additional R285G mutation slightly attenuated the anti-HIV-1 activity of CM TRIMCyp-minor (NE). Residue 285 is in the linker region between the coiled-coil and CypA domains. The precise mechanism of how aa 285 affects anti-HIV-1 activity is unclear at present, but our result was consistent with those of Dietrich *et al.* (2011) and further revealed the importance of a single amino acid substitution at aa 285 on the antiviral activity of CM TRIMCyp.

We showed that a prototypic HIV-1mt, named NL-DT5R, encoding L4/5 of SIVmac239 CA instead of that derived from HIV-1, evaded restriction by the major haplotype of CM TRIMCyp. As only HIV-1-derived L4/5 but not the SIVmac-derived L4/5 is expected to bind to CypA (Franke *et al.*, 1994), the substitution of L4/5 results in loss of binding of the capsid from CypA as well as TRIMCyp. Moreover, we recently demonstrated that HIV-1mt has the ability to grow in CMs (Saito *et al.*, 2011). Retrospective analysis of the *TRIM5* genotypes of the infected CMs revealed that they were homozygous for TRIMCyp (data not shown), suggesting that TRIMCyp homozygotes allow the replication of HIV-1mt *in vivo*. These findings will be helpful not only to understand the molecular mechanisms of the species barrier of primates to lentiviruses, but also to emphasize the importance of *TRIM5* genotypes for future studies regarding non-human primate models for HIV-1 infection.

## Methods

### 

#### Sample collection.

Blood samples were obtained from CMs kept in the Tsukuba Primate Research Center (TPRC), National Institute of Biomedical Innovation, Tsukuba, Japan. CMs have been maintained in indoor facilities as closed colony monkeys in TPRC since 1978 (Honjo, 1985). CMs in TPRC were obtained from Indonesia, Malaysia and the Philippines. Although the detailed local information of their origin is unclear, more than 100 animals were introduced to each colony by dividing it several times. Basically, the monkeys have been bred as pure blood of each origin without interbreed crossing. The generation number of animals involved in this study ranged from two to four when we consider the wild-caught founders (introduced monkeys) as zero. These animals were maintained according to the rules of the National Institute of Biomedical Innovation and guidelines for experimental animal welfare. Bleeding was performed under ketamine hydrochloride anaesthesia.

#### PCR amplification and sequence analysis.

Genomic DNA was extracted from peripheral blood mononuclear cells (PBMCs) of 126 CMs using a QIAamp DNA Blood Mini kit (Qiagen). To test for the *CypA* insertion, the 3′ region of the *TRIM5* gene was amplified by PCR using LA *Taq* (TaKaRa) with primers TC forward (5′-TGACTCTGTGCTCACCAAGCTCTTG-3′) and TC reverse (5′-ACCCTACTATGCAATAAAACATTAG-3′), as described by Wilson *et al.* (2008a). The amplified products of *CypA* from 30 TRIMCyp homozygotes and 32 TRIMCyp/TRIM5α heterozygotes were gel-purified and subjected to direct sequencing using the forward and reverse primers.

To determine the sequences of the RING, B-box, coiled-coil and linker domains of TRIM5α and TRIMCyp, which span >15 kb of genomic DNA, we prepared phytohaemagglutinin (PHA)-stimulated PBMCs from six TRIMCyp homozygotes and three TRIM5α homozygotes. Total RNA was extracted from these cells using TRIzol (Invitrogen), and the RNA was reverse-transcribed using SuperScript III reverse transcriptase (Invitrogen) with TC reverse primer for TRIMCyp or TRIM5 reverse primer (5′-GAATTCTCAAGAGCTTGGTGA-3′) for TRIM5α. The resultant cDNA was then PCR-amplified with LA *Taq* and forward primer TRIM5-235F (5′-GCAGGACCAGTGGAATAGC-3′). The amplified products were purified and subjected to direct sequencing using primers TRIM5-235F, TRIM-N (5′-AGGCAGAAGCAGCAGGAA-3′), TRIM-Nrev (5′-TTCCTGCTGCTTCTGCCT-3′) and TRIM-E (5′-ACCTCCCAGTAATGTTTC-3′). As the direct sequencing results of exons 5 and 6 of TRIMCyp were ambiguous because of the existence of the other splicing variant containing exons 1–4 combined with *CypA* (Brennan *et al.*, 2008), amplified products were then cloned into the vector pCR-2.1TOPO (Invitrogen) and the nucleotide sequences of numerous independent clones (between three and nine) for each TRIMCyp were determined.

Exon 8 (PRYSPRY domain) was PCR-amplified from 12 TRIM5α homozygotes and seven TRIMCyp homozygotes by using TRIMgenotyping forward (5′-CTTCTGAACAAGTTTCCTCCCAG-3′) and reverse (5′-ATGAGATGCACATGGACAAGAGG-3′) primers. The amplified products were purified and subjected to direct sequencing using the TRIM genotyping forward and reverse primers.

#### Cloning and expression of TRIMCyp.

cDNA of the major haplotype of CM TRIMCyp, CM TRIMCyp-major (DK), was amplified by RT-PCR of mRNA extracted from the TRIM5α/TRIMCyp-heterozygous CM T-cell line HSC-F using Not7TRIM5 (5′-GCGGCCGCAGCTACTATGGCTTCTG-3′) as the forward primer (*Not*I site underlined) and CypA Rev (5′-ACGGCGGTCTTTTCATTCGAGTTGTCC-3′) as the reverse primer. RM TRIMCyp cDNA was amplified by RT-PCR of mRNA extracted from the TRIMCyp homozygous RM T-cell line HSR5.4 using Not7TRIM5 as the forward primer and CypA Rev as the reverse primer. The amplified products were then cloned into pCR-2.1TOPO and the authenticity of the nucleotide sequence was verified. To generate TRIMCyp cDNAs carrying a haemagglutinin (HA; YPYDVPDYAA) tag at the C terminus, the TRIMCyp cDNA clones were used as templates for PCR amplification with a primer including a *Not*I site and an HA tag.

To generate the minor haplotype, CM TRIMCyp-minor (NE), the C-terminal portion of RM TRIMCyp (*Sal*I–*Not*I) and the N-terminal portion of CM TRIMCyp-major (DK) (*Not*I–*Sal*I) were assembled in the pcDNA3.1 (−) vector (Invitrogen). CM TRIMCyp-minor R285G was generated by site-directed mutagenesis by a PCR-mediated overlap primer-extension method.

The entire coding sequences of these TRIMCyps were then transferred to the *Not*I site of the pSeV18+b (+) vector. Recombinant SeVs carrying various TRIMCyp were recovered according to a previously described method (Nakayama *et al.*, 2005). The viruses were passaged twice in embryonated chicken eggs and used as stocks for all experiments.

#### Virus propagation.

Virus stocks were prepared by transfection of 293T cells with HIV-1 NL4-3, HIV-2 GH123, SIVmac239 and HIV-1mt NL-DT5R (Kamada *et al.*, 2006) using a calcium phosphate co-precipitation method. Virus titres were measured using p24 (for HIV-1 and HIV-1mt) or p27 (for HIV-2 and SIVmac239) RetroTek antigen ELISA kits (ZeptoMetrix).

#### Virus infection.

Aliquots of 2×10^5^ MT4 cells were infected with SeV expressing CM TRIM5α or each TRIMCyp at an m.o.i. of 10 and incubated at 37 °C for 9 h. Cells were then superinfected with 20 ng HIV-1 NL4-3 or HIV-1mt DT5R p24, 20 ng HIV-2 GH123 p25 or 20 ng SIVmac239 p27. The culture supernatants were collected periodically, and the levels of p24, p25 and p27 were measured with a RetroTek antigen ELISA kit.
